# BTBR *ob/ob* mouse model of type 2 diabetes exhibits early loss of retinal function and retinal inflammation followed by late vascular changes

**DOI:** 10.1007/s00125-018-4696-x

**Published:** 2018-08-09

**Authors:** Vivian K. Lee, Brett M. Hosking, Joanna Holeniewska, Ewa C. Kubala, Peter Lundh von Leithner, Peter J. Gardner, Richard H. Foxton, David T. Shima

**Affiliations:** Translational Vision Research, UCL Institute of Ophthalmology, University College London, 11-43 Bath Street, London, EC1V 9EL UK

**Keywords:** Diabetes, Diabetic retinopathy, Eye, Obesity, Retina, Type 2 diabetes mellitus

## Abstract

**Aims/hypothesis:**

Diabetic retinopathy is increasing in prevalence worldwide and is fast becoming a global epidemic and a leading cause of visual loss. Current therapies are limited, and the development of effective treatments for diabetic retinopathy requires a greater in-depth knowledge of disease progression and suitable modelling of diabetic retinopathy in animals. The aim of this study was to assess the early pathological changes in retinal morphology and neuronal, inflammatory and vascular features consistent with diabetic retinopathy in the *ob/ob* mouse model of type 2 diabetes, to investigate whether features similar to those in human diabetic retinopathy were present.

**Methods:**

Male and female wild-type (+/+), heterozygous (+/−) and homozygous (−/−) BTBR *ob/ob* mice were examined at 6, 10, 15 and 20 weeks of age. Animals were weighed and blood glucose was measured. TUNEL and brain-specific homeobox/POU domain protein 3A (BRN3A) markers were used to examine retinal ganglion cells. We used immunostaining (collagen IV and platelet endothelial cell adhesion molecule [PECAM]/CD31) to reveal retinal vessel degeneration. Spectral domain optical coherence tomography was used to reveal changes in the thickness and structure of the retinal layer. Vitreous fluorophotometry was used to investigate vascular permeability. A-waves, b-waves and oscillatory potentials were measured under photopic and scotopic conditions. Concanavalin A leucostasis and immunostaining with glial fibrillary acidic protein (GFAP) and ionised calcium-binding adapter molecule 1 (IBA-1) identified differences in inflammatory status. Paraffin sections and transmission electron microscopy were used to reveal changes in the thickness and structure of the retinal layer.

**Results:**

Following the development of obesity and hyperglycaemia in 2-week-old and 3-week-old *ob*^−^/*ob*^−^ mice, respectively (*p* < 0.001), early functional deficits (*p* < 0.001) and thinning of the inner retina (*p* < 0.001) were identified. Glial activation, leucostasis (*p* < 0.05) and a shift in microglia/macrophage phenotype were observed before microvascular degeneration (*p* < 0.05) and elevated vascular permeability occurred (*p* < 0.05).

**Conclusions/interpretation:**

The present characterisation of the development of diabetic retinopathy in the *ob/ob* mouse represents a platform that will enable the development of new therapies, particularly for the early stages of disease.

**Electronic supplementary material:**

The online version of this article (10.1007/s00125-018-4696-x) contains peer-reviewed but unedited supplementary material, which is available to authorised users.

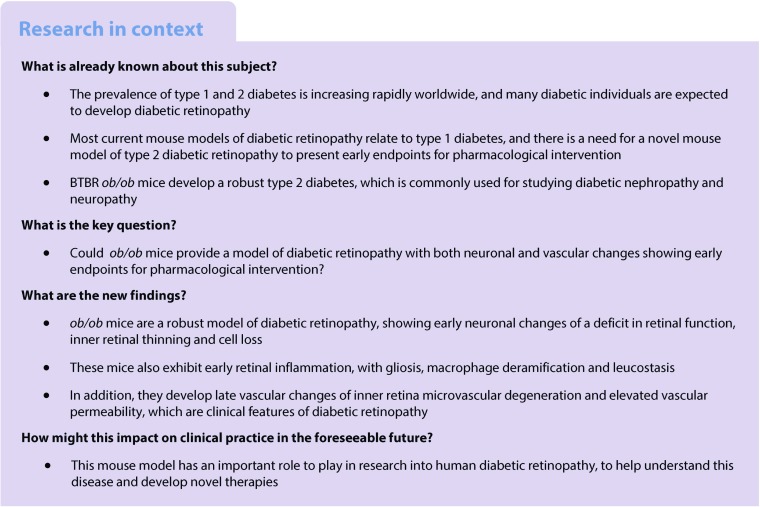



## Introduction

Diabetic retinopathy, which occurs in both type 1 and type 2 diabetes, is fast becoming a worldwide epidemic. The global prevalence of type 2 diabetes is rapidly increasing, and diabetic retinopathy continues to be one of the leading causes of visual loss in adults aged 20–74 years in developed countries [1]. Most individuals with type 1 diabetes develop diabetic retinopathy, and a growing number of those with type 2 diabetes now manifest it, with high blood sugar, insulin resistance and a relative lack of insulin for which limited therapies are currently available.

There are urgent needs to develop both prevention and intervention strategies. One major impediment to this is the lack of a well-characterised model of diabetic retinopathy in type 2 diabetes in which obesity is one of the main traits; such a model could drive a new mechanistic understanding and allow the validation of new therapeutic targets. The BTBR *ob/ob* mouse is a well-established, robust model of diabetic neuropathy [2] and diabetic nephropathy [3] in type 2 diabetes. Published data on these mice at 22 weeks of age suggest that they also develop retinal thinning [4]. We initiated an in-depth characterisation of the retinal phenotype of this model, aiming to describe the earliest pathology and define useful endpoints for pharmacology.

The obese gene (*ob*; also known as *Lep*) encodes the peptide hormone leptin, which is produced mainly from adipocytes to induce satiety. Preclinical studies on rodent models of type 2 diabetes have shown how leptin treatment can regulate glucose homeostasis [5, 6] and reduce body weight and food intake [7]. However, leptin treatment in obese human participants with type 2 diabetes did not have any weight loss effects and only marginally reduced blood glucose [8, 9]. When the *ob* allele of the leptin gene was crossed into the BTBR mouse strain, this BTBR *ob/ob* mouse developed obesity due to a lack of appetite control [10, 11] and manifested characteristics of type 2 diabetes such as progressive insulin resistance, hyperglycaemia and glucose intolerance [12, 13].

The first clinical features of diabetic retinopathy to be recognised are retinal microvascular abnormalities. The initial signs are non-proliferative, such as rupture of blood vessels, capillary dilation dysfunction and microaneurysms. In addition, increased vascular permeability and degeneration are important in the development of retinopathy and visual impairment in diabetes. Furthermore, progression of the retinopathy leads to rosary-like or beading abnormalities of retinal veins [1]. The retinal microvessel network in BTBR *ob/ob* mice has previously been studied using in vivo optical coherence tomography (OCT)/microangiography [4], revealing some similarities with human diabetic retinopathy. These authors concluded that, although the capillary density did not differ from that of wild-type mice and no microaneurysms occurred, retinal blood flow was significantly lower. They also reported that the thickness of the nerve fibre layer/inner plexiform layer (NFL/IPL) was reduced in the same adult mice. This conclusion was supported by another study as an event prior to a reduction in retinal function in the diabetic *db/db* mouse [14]. The retinal thinning occurs as a result of progressive neuronal alterations such as loss of synaptic activity and dendrites, apoptosis of neurons in the IPL and ganglion cells, and activation of microglial cells [15, 16].

The purpose of the current study is to provide an in-depth characterisation of the progression of diabetic retinopathy in BTBR *ob/ob* mice in terms of its vascular, neurodegenerative and inflammatory manifestations. This should prove useful for both elucidating early neurodegenerative disease mechanisms in the retina and providing a platform for preclinical drug discovery.

## Methods

### Animals

Male and female wild-type (*+/+*) and heterozygous (+/−) BTBR *ob/ob* (obese) mice were obtained from the Jackson laboratory (BTBR.Cg-*Lep*^*ob*^/WiscJ; Bar Harbor, ME, USA). These two genotypes and homozygous (−/−) BTBR *ob/ob* mice were bred at the UCL Institute of Ophthalmology. The Jackson Laboratory website reports no correlations between any known retinal degeneration genes and the BTBR mouse strain (www.jax.org/research-and-faculty/tools/eye-mutant-resource/retinal-degeneration-genes, last updated January 2018). Mice received food and water ad libitum, in a 12 h day/night cycle, temperature-controlled, clean environment.

All experiments were conducted according to UK Home Office Guidelines (/www.gov.uk/guidance/research-and-testing-using-animals, accessed Feb 2017) and Association for Research in Vision and Ophthalmology Statement for the Use of Animals in Ophthalmic and Vision Research guidelines (www.arvo.org/About/policies/statement-for-the-use-of-animals-in-ophthalmic-and-vision-research/, last revised September 2016).

### In vivo assays and recovery from anaesthesia

When required, mice were anaesthetised with an intraperitoneal injection of midazolam (Hypnovel; Roche, Welwyn Garden City, UK) 5 mg/kg, medetomidine (Domitor; Orion Pharma, Newbury, UK) 0.5 mg/kg and fentanyl (Sublimaze; Janssen, High Wycombe, UK) 0.05 mg/kg in water. Their pupils were dilated with phenylephrine hydrochloride 2.5% wt/vol. and tropicamide 1% wt/vol. (Bausch and Lomb, Kingston upon Thames, UK) before in vivo imaging or electroretinography was undertaken. Afterwards, mice were brought round with naloxone (Hameln, Gloucester, UK) 1.2 mg/kg, atipamezole (Antisedan; Orion Pharma) 2.5 mg/kg and flumazenil (Hameln) 2.5 mg/kg in saline (154 mmol/l NaCl).

The three genotypes were evaluated at 6, 10, 15 and 20 weeks of age. The animals were weighed, and blood glucose concentrations were measured (AlphaTRAK glucometer; Alameda, Pompano, FL, USA) at the same time of the day for all the animals in the study, at each of the ages. All in vivo studies (electroretinograms, leucostasis and OCT) except for fluorophotometry were carried out at these ages. Photographs of the physical appearances of mice were captured at 6 and 20 weeks of age.

### Immunohistochemistry

To collect cross sections, eyes were fixed in 4% wt/vol. paraformaldehyde for 1 h at room temperature and prepared as transverse sections. Eyes were cryoprotected in 30% wt/vol. sucrose and snap-frozen in an optimum cutting temperature compound (Tissue-Tek; Sakura Finetek, Thatcham, UK). Cryostat sections (10 μm) were then thaw-mounted onto glass slides. Sections were blocked for 1 h with 5% wt/vol. normal goat serum and permeabilised in 0.3% wt/vol. Triton X-100 in PBS (T-PBS). They were then incubated overnight with primary antibodies (Table 1) in blocking solution at room temperature. Secondary antibodies were applied for 1 h at room temperature, and the sections stained with 5 μmol/l DAPI. After the final washes, the tissue sections were mounted in Vectashield (Vector Laboratories, Peterborough, UK) and covered with a cover slip.

**Table 1:** List of antibodies for immunohistochemistry

Primary antibodies			Corresponding secondary antibodies^a^	
Antibody	Type and dilution	Manufacturer	Antibody	Type
Rabbit anti-collagen IV	Polyclonal 1:500	Bio-Rad, Hercules, CA, USA	Donkey anti-rabbit conjugated to Alexa Fluor 488 nm	Polyclonal
Rat anti-PECAM/CD31	Monoclonal 1:500	BD Biosciences, San Jose, CA, USA	Donkey anti-rat conjugated to Alexa Fluor 647 nm	Polyclonal
Goat anti-IBA-1	Polyclonal 1:500	Abcam, Cambridge, UK	Donkey anti-goat conjugated to Alexa Fluor 488 nm	Polyclonal
Rabbit anti-GFAP	Polyclonal 1:500	Dako, Stockport, UK	Donkey anti-rabbit conjugated to Alexa Fluor 594 nm/488 nm	Polyclonal
Goat anti-BRN3A	Polyclonal 1:200	Millipore, Watford, UK	Donkey anti-goat conjugated to Alexa Fluor 488 nm	Polyclonal

^a^All 1:1000; Thermo Fisher, Paisley, UK

For retinal whole-mounts, animals were euthanised and their retinas were dissected out and fixed as described above. Whole-mount preparations were blocked for 2 h in 5% wt/vol. donkey serum and permeabilised in 3% wt/vol. T-PBS for 2 h at room temperature. They were then incubated with primary antibodies (Table 1) in blocking solution overnight at room temperature. Secondary antibodies were applied for 2 h at room temperature and sections prepared as described above.

The antibodies were used as per the manufacturers’ instructions. The negative control procedures involved no primary antibody and relevant IgG isotypes. Images of sections or retinas were taken on Zeiss 700 or 710 confocal microscopes (Carl Zeiss, Cambridge, UK).

### Leucostasis

Concanavalin A leucostasis was performed as described by Joussen et al [17]. After inducing deep anaesthesia with 200 mg/kg pentobarbital sodium (Euthatal, Merial, Harlow, UK), the mice were cardiac perfused with rhodamine-conjugated concanavalin A (Rho-Con A; Vector Laboratories), followed by PBS. Eyes were fixed in 4% wt/vol. paraformaldehyde for 1 h at room temperature, and the retinas were then mounted whole in Vectashield. Leucocytes inside vessels over the entire retina were counted under an epifluorescence Olympus BX51 microscope with a Retiga 2000R camera (QImaging, Surrey, BC, Canada).

### Preparations of semi-thin optic nerve sections

Optic nerves were processed as previously described by Foxton et al [18]. Briefly, the tissues were fixed overnight in Karnovsky’s solution at 4°C. Specimens were osmicated for 2 h in 1% wt/vol. osmium tetroxide and then dehydrated in 100% ethanol. Next, the optic nerves were incubated in propylene oxide for 30 min and placed in a 50:50 mixture of propylene oxide:Araldite (Electron Microscopy Sciences, Hatfield, PA, USA) overnight. This solution was changed to 100% Araldite wt/vol. and incubated overnight at 60°C. Semi-thin (0.75 μm) sections were cut and stained with 1% wt/vol. toluidine blue/borax in 50% ethanol before examination by light microscopy. For quantification, three non-overlapping images were taken at ×60 magnification, at the centre, middle and periphery of the optic nerve, using an Olympus BX51 microscope with a Retiga 2000R camera (QImaging). The number of axons in each image was counted, averaged for the mean for each optic nerve, and then expressed as axons density per image area (4500 mm^2^).

### Electroretinography

Image-guided large-field scotopic and large-field photopic electroretinograms were recorded from dark- (12 h) and light-adapted mice (Micron IV; Phoenix Research Laboratories, Pleasanton, CA, USA). Scotopic recordings were performed under dim red light. Series of 5 ms single-flash recordings were obtained at increasing light intensities from −2.5 to 3.0 log(cd × s/m^2^), where the logarithmic base is 10. Twenty responses per intensity were averaged for the mean with an interstimulus interval of 20 s. The a-wave, b-wave and oscillatory potential amplitudes were evaluated (LabScribe; iWorx System, Dover, NH, USA).

### Paraffin sections and H&E staining

Retinal thickness was measured from paraffin sections. The eyes were enucleated and fixed in 10% wt/vol. neutral buffered formalin and then processed as described in Table 2.

**Table 2:** Paraffin section processing

Station	Solution	Duration
1	Neutral buffered formalin	1 h
2	70% IDA	1 h
3	90% IDA	1 h
4–7	Absolute IDA	1.5 h each
8–11	Xylene	1 h each
12–14	Paraffin wax (Leica)	1.5 h each

Samples were orientated and embedded in paraffin wax using a Leica Histocenter (Leica, Milton Keynes UK). Once cooled and solidified, they were cut at a thickness of 4 μm and heated for 20 min at 60°C prior to dewaxing. Sections were dewaxed twice for 1 min in xylene and rehydrated by two 1 min exposures to industrial denatured alcohol (IDA; Genta, Rudgate, UK). Following a 30 s wash in running tap water, the slides were stained with H&E using standard protocols.

### Transmission electron microscopy

Retinal morphology was examined using transmission electron microscopy (TEM). Eyeballs were removed and processed as described above, and cut into ultrathin (75 nm) sections. They were then stained with Reynold’s lead citrate and washed in distilled water. The images were captured on a Jeol 1010 microscope (JEOL, Peabody, MA, USA) at 80 kV using DigitalMicrograph software version 3 (Gatan, Abingdon, UK).

### TUNEL assay

The protocol used for the TUNEL assay has previously been described in Foxton et al [18]. TUNEL staining was carried out on whole-mounted tissue. Retinas were dissected out and permeabilised in 3% wt/vol. T-PBS for 2 h at room temperature. The TUNEL protocol was performed according to the manufacturer’s instructions (Promega, Southampton, UK), and the retinas were then stained with 1:500 biotinylated isolectin B4 (IB4; Sigma-Aldrich, Dorset, UK) overnight at 4°C, followed by 1:500 streptavidin labelled with Alexa Fluor 594 for 2 h at room temperature. The tissues were washed in 0.3% wt/vol. T-PBS with 5 mmol/l DAPI and flat-mounted in Vectashield.

To quantify TUNEL-positive neurons, we used a Zeiss 710 confocal microscope (Zeiss, Oberkochen, Germany), generating tiled scans of the entire retina, with 30 μm Z-stacks through the ganglion cell layer (GCL) using a ×10 magnification water-immersion objective. IB4 staining and morphological criteria discriminated non-neuronal (endothelial and glial) cells from neuronal cells. The total number of TUNEL-positive cells in the GCL was counted.

### Spectral domain OCT

The spectral domain OCT (SD-OCT) studies were performed using a Bioptigen Envisu R2200 SD-OCT imaging system (Bioptigen, Morrisville, NC, USA). Mice were anaesthetised and their pupils dilated as described above. Rectangular scans were performed, consisting of a 1.4 mm × 1.4 mm perimeter with 1000 A-scans per B-scan, with a total of 100 B-scans. Scans were obtained after localising the optic nerve. InVivoVue Reader version 2 (Bioptigen) was used to analyse the scans, and measurements were performed 500 μm away from the optic disc. The thicknesses of the following layers were measured: ONL, outer plexiform layer (OPL), inner nuclear layer (INL), a complex comprising the IPL, GCL and NFL, which we called IPL+GCL+NFL, and all the inner retinal layers together, which included the OPL, INL and IPL+GCL+NFL.

### Fluorophotometry

For this we used an ocular fluorophotometer, the Fluorotron Master Research Mouse Edition, which was kindly provided by Ocumetrics (Mountain View, CA, USA) for a short-term trial. Mice were anaesthetised and their pupils dilated as described above. For each animal, 50 μl of 1% wt/vol. (500 μg) fluorescein (Martindale, Romford, UK) was administered intravenously into a tail vein. After 10 min of administration, both eyes were scanned at four steps per millimetre. The fluorogram peak corresponding to the vitreous body was used. The readings were corrected against measurements of plasma fluorescein using cuvettes with the same fluorophotometer.

### Statistical analysis

Randomisation was not carried out on the three genotypes during in vivo experiments because of the nature of animal breeding. Where possible, the experimenters were masked during the assessment and analysis, for example in the ex vivo experiments. Data are expressed as means and error bars are SEM. No criteria were applied to exclude data from the statistical analysis. Statistical analyses were performed using GraphPad Prism software version 6 (GraphPad Software, La Jolla, CA, USA). For comparisons between two groups, such as the 6- and 20-week-old animals, or between control and diabetic animals, the Mann–Whitney *U* test was used; the Shapiro–Wilk test was also used to test whether the data were normally distributed. The leucostasis, INL/ONL nuclear count, TUNEL assay, brain-specific homeobox/POU domain protein 3A (BRN3A) cell count, platelet endothelial cell adhesion molecule (PECAM, also known as CD31) and collagen IV count and fluorophotometry data were analysed in this way. When comparing longitudinal data (weight, blood glucose, electroretinogram and SD-OCT data), one-/two-way ANOVA and Newman–Keuls post hoc test were used.

## Results

### Obesity and hyperglycaemia

**Fig. 1:**
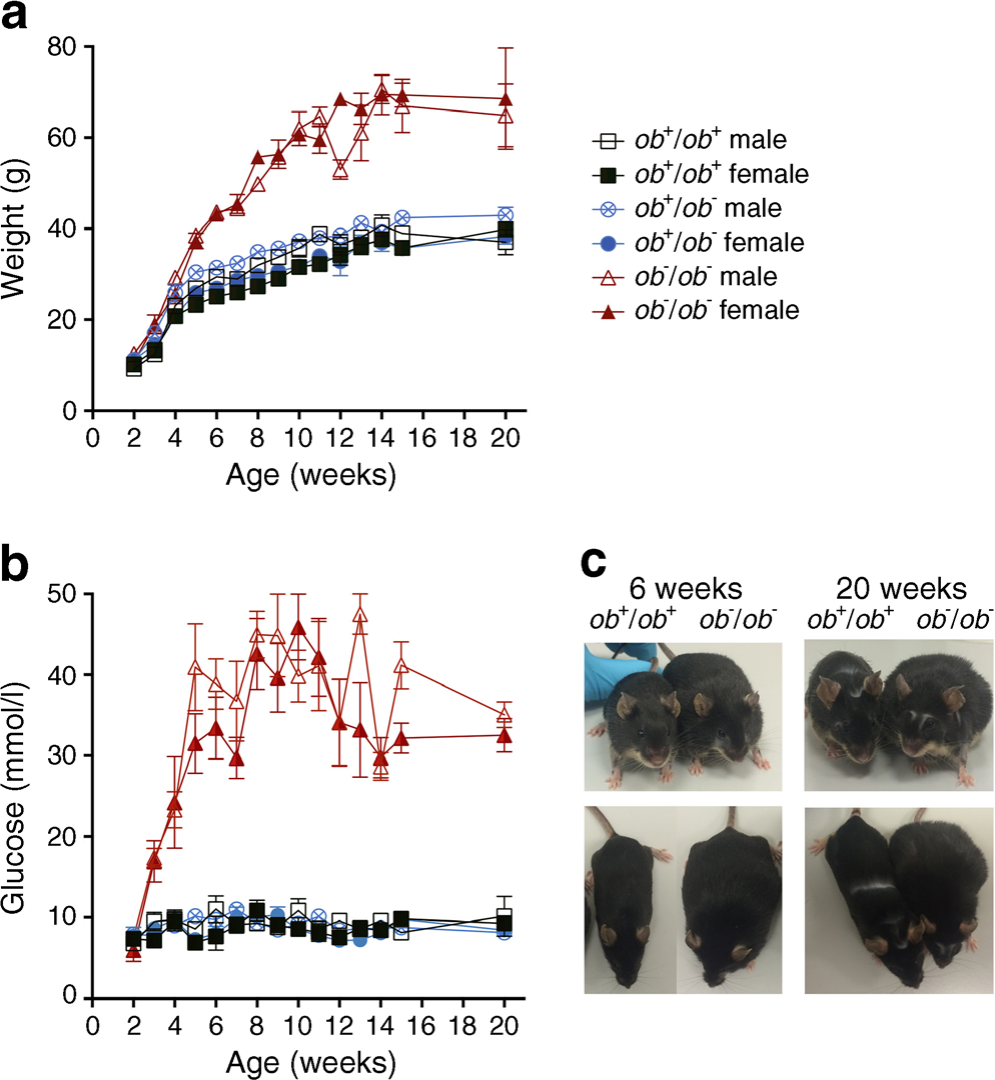
Body weight (**a**) and blood glucose levels (**b**) of all mouse phenotypes from 2 to 20 weeks of age. At 2 weeks of age, the body weights of both male and female *ob*^−^/*ob*^−^ mice were significantly greater than those of *ob*^+^/*ob*^+^ and *ob*^+^/*ob*^−^ controls (unpaired *t* test *ob*^+^/*ob*^+^ and *ob*^+^/*ob*^−^ vs *ob*^−^/*ob*^−^, two-tailed, *p* < 0.05). Blood glucose measurements showed that 3-week-old male and female *ob*^−^/*ob*^−^ mice exhibited an early onset of hyperglycaemia. Data are mean ± SEM. Both datasets in (**a**) and (**b**) were tested with one-way ANOVA, *n* = 6 animals; *p* < 0.001. The key next to (**a**) applies to both graphs. (**c**) Images showing the appearance of the *ob*^+^/*ob*^+^ and *ob*^−^/*ob*^−^ mice at 6 and 20 weeks of age showed almost a doubling of girth in 6-week-old *ob*^−^/*ob*^−^ mice in comparison to *ob*^+^/*ob*^+^ mice. The weight accumulated around the lower abdomen, as seen in the older (20-week-old) animals. With ageing, the fur around the eye and nose, in the mid-back and on the lower area of the front was gradually lost

The lack of appetite control in the leptin-deficient BTBR *ob*^−^/*ob*^−^ mice led to an increase in both body weight and degree of hyperglycaemia over time. The body weight and blood glucose concentrations of these mice were monitored to determine the rate at which they progressed into obesity and diabetes. The weight of the *ob*^−^/*ob*^−^ mice began to significantly separate from those of the *ob*^+^/*ob*^+^ and *ob*^+^/*ob*^−^ mice at 2 weeks of age, so this age was considered to represent the onset of obesity (Fig. 1a). Whereas the *ob*^+^/*ob*^+^ and *ob*^+^/*ob*^−^ mice maintained their adult body weight at a mean of 23–39 g, the *ob*^−^/*ob*^−^ mice continued to gain weight up to at least 20 weeks of age, reaching a maximum mean weight of 64–69 g.

We considered the mice to be hyperglycaemic when their blood glucose level increased above 13 mmol/l. The *ob*^−^/*ob*^−^ mice reached this level at 3 weeks of age (Fig. 1b). At 20 weeks of age, the blood glucose level was two- to fivefold greater than that of *ob*^+^/*ob*^+^ and *ob*^+^/*ob*^−^ mice. Thus, the onset of hyperglycaemia occurred approximately 1 week after the onset of obesity. The onsets of these changes were similar between sexes and the two conditions persisted with age; therefore the effect of sex on the following endpoints was not differentiated. The physical appearance of the mice differed according to weight (Fig. 1c).

### Inflammation

**Fig. 2:**
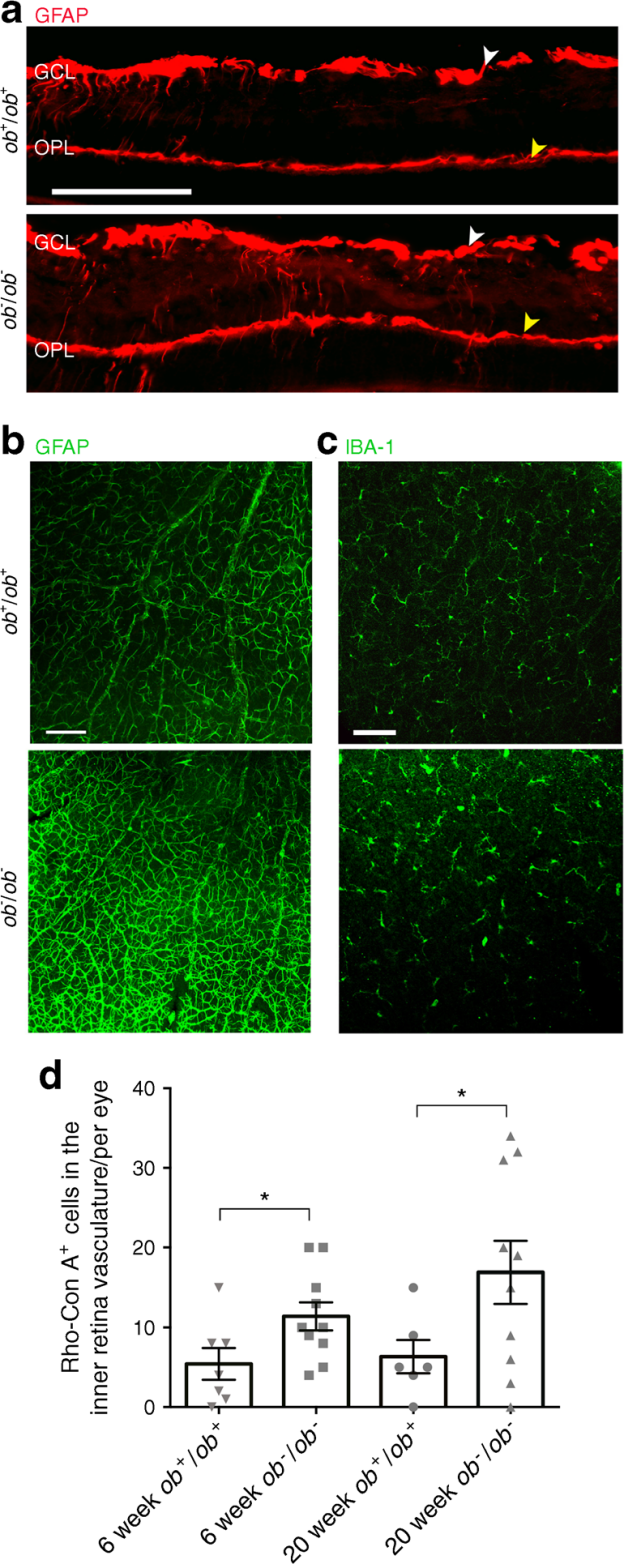
(**a**) GFAP is found along the GCL, and Müller cell processes are seen to project towards the plexiform layer. Representative images of GFAP expression (red) in transverse sections of eyes from 6-week-old *ob*^−^/*ob*^−^ and *ob*^+^/*ob*^+^ mice reveal elevated GFAP expression in *ob*^−^/*ob*^−^ mice along the GCL (white arrowheads) and OPL (yellow arrowheads), suggesting gliosis. Scale bar, 100 μm. (**b**) In the retinal whole-mounts, there were more GFAP-labelled Müller cells (green) in the primary plexus layer in *ob*^−^/*ob*^−^ than *ob*^+^/*ob*^+^ mice. Gliosis by upregulated Müller cells reflects inflammation of the nervous system. Scale bar, 50 μm. (**c**) In the same layer of retinal whole-mounts, the IBA-1-labelled macrophages (green) were more abundant, amoeboid and activated in *ob*^−^/*ob*^−^ mice. Scale bars, 150 μm. (**d**) Graph of the number of Rho-Con A-labelled leucocytes in the main retinal blood vessels in each eye, in 6- and 20-week-old *ob*^+^/*ob*^+^ and *ob*^−^/*ob*^−^ mice. In both age groups, there were more leucocytes in the eyes of *ob*^−^/*ob*^−^ than *ob*^+^/*ob*^+^ mice. This was significant at both 6 weeks (*n* = 6 eyes) and 20 weeks (*n* = 7 eyes); *p < 0.05 for comparisons shown, Mann–Whitney *U* test, one-tailed. Data are mean ± SEM

Immunocytochemical examination of the eyes in young mice (6 weeks of age) revealed signs of inflammation before the commonly observed features of diabetic retinopathy. Staining for glial fibrillary acid protein (GFAP) revealed glial activation during the development of diabetic retinopathy, with increased expression along the GCL and primary plexus (Fig. 2a) and the appearance of a more stellate phenotype with age (Fig. 2b). Although the expression and level of GFAP were not uniform across the retina, we nevertheless observed increased GFAP staining in the GCL, with more frequent and deeper Müller cell processes in the diabetic retina. The use of the microglia/macrophage marker ionised calcium-binding adapter molecule 1 (IBA-1) highlighted the deramification of the macrophages—from dendritic to amoeboid morphology with larger cell bodies—in the eyes of diabetic mice (Fig. 2c). The number of adherent leucocytes observed by perfusion of Rho-Con A into the retinal vessels (see the electronic supplementary material [ESM] Fig. 1a) increased more than twofold in 6-week-old diabetic mice compared with age-matched wild-type mice, and threefold at age 20 weeks (Fig. 2d).

### Retinal electrophysiology

**Fig. 3:**
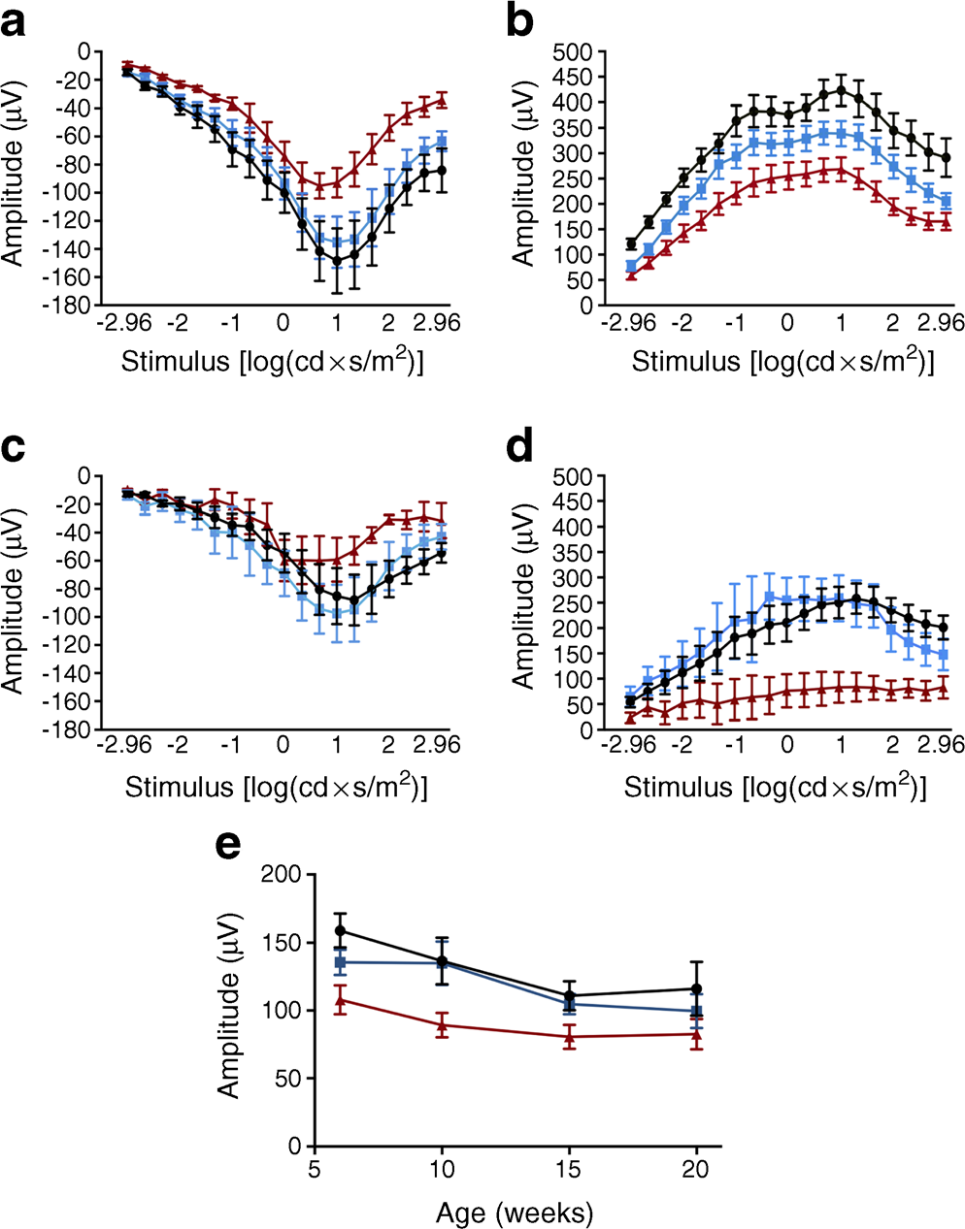
Electroretinography was performed to show the functioning of the retinal neuronal cells. There was a loss of retinal function in the *ob*^−^/*ob*^−^ mice in comparison to the healthy *ob*^+^/*ob*^+^ and *ob*^+^/*ob*^−^ mice. Scotopic electroretinogram showing a-waves (**a**) and b-waves (**b**) in 6-week-old mice, and a-waves (**c**) and b-waves (**d**) in 20-week-old mice. The amplitudes of the a-waves, originating from the responses of the rod and cone photoreceptors, were reduced in the retinas from both 6- and 20-week-old *ob*^−^/*ob*^−^ mice. The amplitude of the b-wave represents responses from cells in the inner retina, for example bipolar cells, and the same reductions in amplitude were seen in *ob*^−^/*ob*^−^ mice. (**e**) Under photopic conditions, the b-wave responses were most different when the maximum light level [2.87 log(cd × s/m^2^)] was used. The *ob*^−^/*ob*^−^ animals consistently showed a reduced response at 6, 10, 15 and 20 weeks of age. Overall, the electroretinogram recordings from *ob*^−^/*ob*^−^ retina were weaker than in age-matched *ob*^+^/*ob*^+^ and *ob*^+^/*ob*^−^ mice between 6 and 20 weeks of age. Two-way ANOVA: age, *p* < 0.001; phenotype, *p* < 0.001 for all figure parts (**a**–**e**). Data are mean ± SEM, *n* = 12 eyes. Black circles, *ob*^+^/*ob*^+^; blue squares *ob*^+^/*ob*^−^; red triangles *ob*^−^/*ob*^−^

Early loss of retinal function was shown using electroretinograms in 6-week-old mice. The eyes were subjected to increasing light stimuli under either scotopic (Fig. 3a–d) or photopic (Fig. 3e) conditions. The photoreceptor responses from the three mouse phenotypes are seen in the a-waves of the scotopic electroretinogram (Fig. 3a, c). The responses were weaker in retina from *ob*^−^/*ob*^−^ mice compared with *ob*^+^/*ob*^+^ (−31%) or *ob*^+^/*ob*^−^ (−37%) animals, even in 6-week-old mice (Fig. 3a). The b-waves are responses from the inner retina, such as from bipolar cells, and were also weaker in the young *ob*^−^/*ob*^−^ retina compared with age-matched *ob*^+^/*ob*^+^ (−37%) and *ob*^+^/*ob*^−^ (−20%) retina (Fig. 3b). Even in non-diabetic animals, these amplitudes generally decreased with age, suggesting that retinal function worsens. Under photopic conditions (Fig. 3e), b-wave responses were investigated using the maximum light level [2.87 log(cd × s/m^2^)]. The *ob*^−^/*ob*^−^ animals consistently showed a reduced response between 6 and 20 weeks of age. These results were also seen in the oscillatory potential (ESM Fig. 2a).

### SD-OCT

**Fig. 4:**
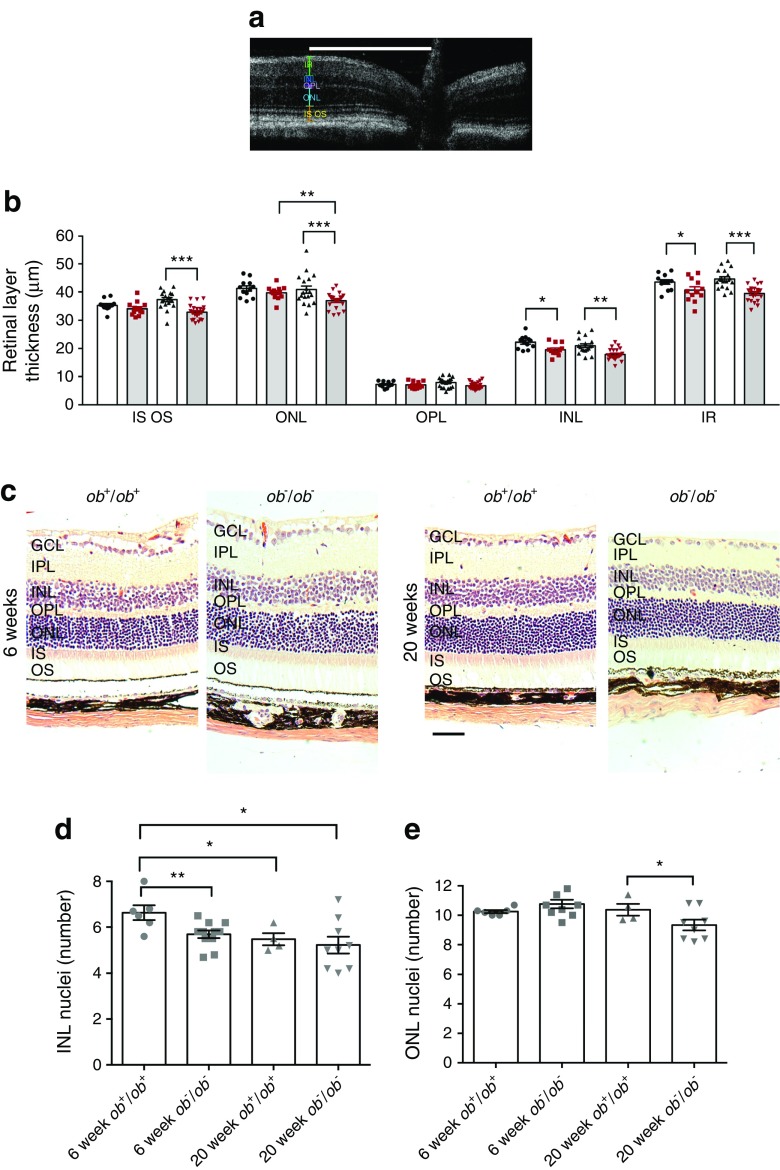
(**a**) Representative image of 6-week-old *ob*^+^/*ob*^+^ retina captured using SD-OCT showing the different retinal layer thicknesses. Scale bar, 250 μm. IR, inner retina, IS, inner segment; OS outer segment. (**b**) Quantification of 12 images from each phenotype are represented in the bar graph. The thickness of the outer retinal layer was similar between the 6-week-old control and diabetic retina, but that of the INL and IR was reduced. This was exacerbated at 20 weeks of age, with the eyes of diabetic mice showing a reduction in thickness in the IS and OS layer, ONL, INL and IR. In particular, the ONL in diabetic mice was significantly reduced with age compared with non-diabetic mice. Two-way ANOVA: retinal layers, *p* < 0.001; phenotype, *p* < 0.001. *p < 0.05, **p < 0.01, ***p < 0.001 for comparisons shown, Mann–Whitney *U* test, one-tailed. Data are mean ± SEM, *n* = 12 eyes. Black circles, 6 week *ob*^+^/*ob*^+^; red squares, 6 week *ob*^−^/*ob*^−^; black triangles, 20 week *ob*^+^/*ob*^+^; red inverted triangles, 20 week *ob*^−^/*ob*^−^. (**c**) Representative H&E-stained, paraffin-embedded transverse retinal sections of 6- and 20-week-old *ob*^+^/*ob*^+^ and *ob*^−^/*ob*^−^ mice. The histology was similar in the 6-week-old *ob*^+^/*ob*^+^ and *ob*^−^/*ob*^−^ mice. However, in 20-week-old mice, the retinal layers were thinner in the *ob*^−^/*ob*^−^ mice. Scale bar, 50 μm. (**d**) Number of INL nuclei stacked in 6- and 20-week-old *ob*^+^/*ob*^+^ and *ob*^−^/*ob*^−^ mice. The data suggest that there were fewer cells in this layer in the young diabetic mice and that the number continued to decrease with age (*ob*^+^/*ob*^+^; *ob*^−^/*ob*^−^). (**e**) Number of ONL nuclei in the same animals, suggesting that there was a slight loss of photoreceptors in the aged diabetic mice. *p < 0.05, **p < 0.01 for comparisons shown, Mann–Whitney *U* test, one-tailed. Data are mean ± SEM, *n* = 6 eyes

Measurements of retinal layer thickness using SD-OCT in 6- and 20-week-old diabetic animals showed thinning of the retina with age (Fig. 4a, b). The differences in retinal thickness were more evident between the 20-week-old *ob*^+^/*ob*^+^ and *ob*^−^/*ob*^−^ animals. In these older animals, nearly all measurements taken in the *ob*^−^/*ob*^−^ animals showed a significant reduction in thickness of the retinal layer, inner and outer segments, ONL, INL and inner retina. Furthermore, counts of numbers of nuclei in the two nuclear layers in the retina suggested that the greatest reduction of cells was seen in the inner retina (Fig. 4c–e).

### Apoptosis assay

**Fig. 5:**
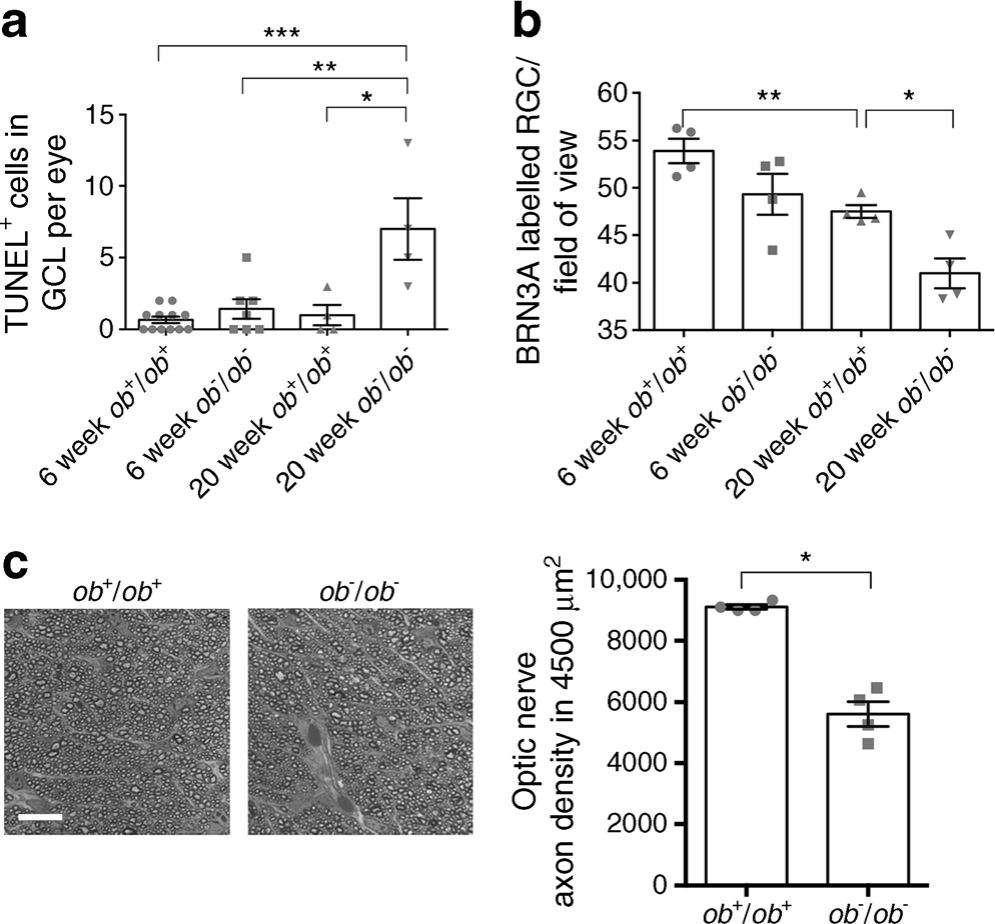
(**a**) Cell count of TUNEL-stained apoptotic cells in the retina of 6- and 20-week-old mice. There were over threefold more TUNEL-positive nuclei in the GCL in 20-week-old *ob*^−^/*ob*^−^ than 6-week-old *ob*^+^/*ob*^+^, 6-week-old *ob*^−^/*ob*^−^ or 20-week-old *ob*^+^/*ob*^+^ mice (*p < 0.05, **p < 0.01, ***p < 0.001 for comparisons shown, Mann–Whitney *U* test, one-tailed). One-way ANOVA, *p* = 0.0001; *n* = 4 eyes. (**b**) Cell counts of BRN3A-stained RGC cells in the GCL in each field of view with an area of 0.65 mm^2^. The number of ganglion cells decreased not only from 6 to 20 weeks, but also with hyperglycaemia between 20-week-old *ob*^+^/*ob*^+^ and *ob*^−^/*ob*^−^ mice (*p < 0.05, **p < 0.01 for comparisons shown, Mann–Whitney *U* test, one-tailed); *n* = 4 eyes. (**c**) The brightfield images and quantification in the bar chart show that optic nerve axon density in RGC cells in the 6-week-old *ob*^−^/*ob*^−^ mice is reduced by approximately one-third (*p < 0.05, Mann–Whitney *U* test, one tailed). The axons from the *ob*^−^/*ob*^−^ eyes are more spread apart between supporting cells compared with those from *ob*^+^/*ob*^+^ eyes. Scale bar, 5 μm. Data are mean ± SEM*, n* = 4 nerves

Retinal whole-mount staining using TUNEL as a marker for cell death (ESM Fig. 3a) revealed that the number of apoptotic cells in the GCL was significantly greater in 20-week-old *ob*^−^/*ob*^−^ mice than age-matched *ob*^+^/*ob*^+^ mice, compared with younger 6 week *ob*^−^/*ob*^−^ and *ob*^+^/*ob*^+^ mice (Fig. 5a). Specific staining of the retinal ganglion cell (RGC) marker using BRN3A revealed that the number of these cells declined significantly in the presence of diabetes and with age (Fig. 5b, ESM Fig. 3b). The axons of the RGCs that formed the optic nerve were examined. The axon density captured from semi-thin sections of the optic nerve revealed a significant reduction in number of these axons, which occurred as early as 6 weeks of age in diabetic mice compared with wild-type controls. By close examination of the IPL using TEM, the axons in aged diabetic retinas appeared either to be fused together or to have lost their cell membrane structures (ESM Fig. 4a).

### Retinal vessel degeneration

**Fig. 6:**
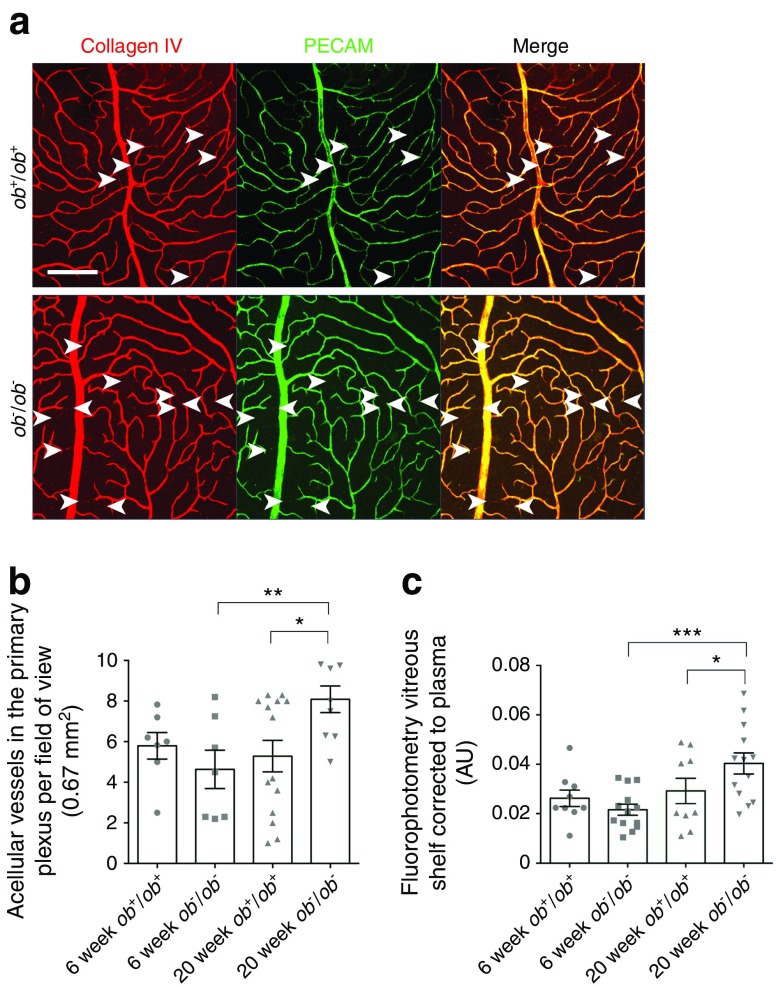
With ageing, there is an increase in some physiological processes, such as blood vessel regression. (**a**) Representative images showing expression of collagen IV (red) and PECAM (green) in the retinal vascular primary plexus of 20-week-old *ob*^−^/*ob*^−^ and *ob*^+^/*ob*^+^ mice. Collagen IV labels the structural scaffolding of the blood vessels, and PECAM labels endothelial cells. The white arrowheads point to microvascular degeneration where only the blood vessel scaffolding remained and the endothelial cells were absent. These regressed vessels were more frequently seen in *ob*^−^/*ob*^−^ than *ob*^+^/*ob*^+^ mice. Scale bar, 50 μm. (**b**) Collagen IV-stained vessels not showing co-localisation of PECAM in the primary plexus were counted from six images captured from the intermediate region of each retina in 6- and 20-week-old *ob*^+^/*ob*^+^ and *ob*^−^/*ob*^−^ mice. There were no significant differences between the two groups of 6-week-old mice, or between the *ob*^+^/*ob*^+^ retinas with age. However, there was significantly more microvascular degeneration in the 20-week-old *ob*^−^/*ob*^−^ retina compared with the 6-week-old *ob*^−^/*ob*^−^ retina and with the aged-matched wild-type retina; *n* = 7 eyes. (**c**) Retinal vessel leakage in the vitreous body was detected using fluorescein fluorophotometry in 6- and 20-week-old *ob*^+^/*ob*^+^ and *ob*^−^/*ob*^−^ mice. There were no significant differences in the *ob*^+^/*ob*^+^ mice with age. In the diabetic mice, however, there were significant changes with age (*n* = 13 eyes). Retinal vascular leakage in older diabetic mice was significantly greater than in age-matched controls (*n* = 9 eyes). *p < 0.05, **p < 0.01, ***p < 0.001 for comparisons shown, Mann–Whitney *U* test, one-tailed. Data are mean ± SEM

Although inflammatory and neuronal factors are believed to drive the early manifestations of diabetic retinopathy, the earliest clinical signs and hallmarks of this disease are related to vascular damage. Microvascular damage such as loss of endothelial cells and increased vascular permeability was observed. We demonstrated a loss of endothelial cells by co-staining these cells (PECAM) with the basement membrane (collagen IV), to identify vascular degeneration in the primary plexus of the retina [19]. Considerably more vessels showed degeneration in the aged *ob*^−^/*ob*^−^ mice than in aged *ob*^+^/*ob*^+^ mice, and also than in aged-matched controls (Fig. 6a, b). There were no significant differences between the groups of 6-week-old mice, or between retinas from young and older *ob*^+^/*ob*^+^ mice.

### Fluorescein fluorophotometry

Vitreous fluorophotometry has been shown to reveal early signs of blood–retinal barrier breakdown [20–22]. Measurements from the vitreous body can highlight vascular leakage in the retina [23]. Vascular permeability determined using fluorescein fluorophotometry indicated that the aged *ob*^−^/*ob*^−^ animals in particular had abnormal, exudative blood vessels (Fig. 6c). There were significant changes between the young and old diabetic mice, but no changes in the *ob*^+^/*ob*^+^ mice. Retinal vascular leakage in older diabetic mice was significantly greater than in aged-matched controls.

## Discussion

We describe here a detailed analysis of retinopathy in a mouse model of type 2 diabetes and obesity, which has shown pathological features consistent with human diabetic retinopathy. These included retinal microvascular changes together with preceding early retinal neurodegeneration and inflammation.

Until now, there has not been a thorough documentation and characterisation of the progression of retinal pathology in type 2 diabetes associated with chronic obesity and insulin insensitivity. This is due in part to the lack of rodent models that can reflect the chronic pathology of human diabetic disease in a time frame suitable for reproduction in laboratory studies. The mouse model of type 2 diabetes and diabetic retinopathy that we describe here shows a relatively early onset of diabetes; in our model retinal functional deficit and inflammatory changes occur at 6 weeks of age. In comparison, other genetic mouse models of diabetic retinopathy, such as Ins2^Akita^ and NOD mice, which are models of type 1 diabetes, show an onset of disease at 8 and 12 weeks of age, respectively [24–26]. Our model, therefore, presents an opportunity to study aspects of early retinal changes and progression of retinopathy that appear to be consistent with human diabetic retinopathy.

Obese mice lacking appetite control due to a genetic deficiency related to the hormone leptin (whether in the receptor or the ligand), which therefore affects its role in the insulin–glucose axis, have been studied with regard to the underlying pathology in diabetic tissue [2], including the retina [4, 14], revealing some of the pathological features of human diabetic retinopathy. However, although leptin receptor-deficient (*db*/*db*) mice have been evaluated for diabetic retinopathy, an extensive characterisation of retinal disease in BTBR *ob/ob* leptin (ligand)-deficient obese mice has not yet been carried out. In comparison with *db/db* mice, *ob/ob* mice exhibit more than fourfold higher blood glucose levels than non-diabetic controls. Similar to *db/db* mice, *ob/ob* mice also show retinal function deficit, loss of ganglion cells, increased apoptotic cells in the same layer and upregulation of GFAP in Müller cells [14]. In terms of measurement of retinal thickness using SD-OCT, our findings support those of other groups using the same animals but at an older, 22 week, time point [4]. The pathological features we report here, such as neuronal dysfunction and loss, gliosis and para-inflammation with retinal leucostasis, microvascular changes and leakage, not only support already-published findings from other animal models of diabetes [4, 14], but can also be considered to resemble many changes that are common to human pathology [1, 27, 28].

A particularly important consideration with regard to correctly interpreting the mechanisms of diabetic retinopathy underlying mouse models of diabetic retinopathy is the combination, in our study, of the BTBR genetic background and leptin deficiency. Although it is known that the BTBR background leads to diabetes in part because it harbours alleles that promote insulin resistance and restrict hepatic lipogenic capacity [12], the BTBR strain is also known to have a heightened, more reactive immune profile [29]. This may explain the loss of retinal function and inflammatory changes we observed from an early stage of the development of diabetes. This manifests as an increased baseline expression of proinflammatory cytokine in the brain and an increased proportion of activated brain microglia [29, 30]. This may explain our observation of a shift in the pattern or phenotype of IBA-1-labelled cells in the inner retina. Other groups have shown that dipeptidyl peptidase-4 (DPP4) inhibitors, which inhibit the degradation of endogenous glucagon-like peptide-1 (GLP-1), attenuate the production of proinflammatory cytokines and lower blood glucose in these mice, suggesting that the inflammatory process could be a promising therapeutic target [31]. In other models of diabetic retinopathy, retinal leucostasis has been observed within days of the onset of diabetes, and was associated with damaged endothelial cells [17]. This is supported in our study by the early Rho-Con A counts, leucostasis being shown to be twofold higher in diabetic mice than controls. The impact of this heightened inflammatory state on neuronal loss following increased glucose levels in the retina requires investigation.

In addition, it is known that the loss of presynaptic protein hinders synaptic function [32], leading to impaired neuron-to-neuron transmission, which could explain the deficit in retinal function and axon abnormalities that we observed in the inner retina. This could be related to elevated GFAP expression, as reported here and previously [33], but the trigger for neurodegeneration could be complex and stem from the malfunction of many pathways.

A key feature of diabetic retinopathy in BTBR *ob/ob* mice is the microvascular changes, including vascular leakage and capillary endothelial cell loss—both hallmarks of human diabetic retinopathy [28]. This aspect is significant given the long-standing evidence, from models of diabetic retinopathy in type 1 diabetes, that retinal vascular changes may be independent of neuronal damage [33]. The BTBR *ob/ob* model of type 2 diabetes develops the same changes observed in models of type 1 diabetes. The similarity of lesions observed in this model to those previously reported in models of type 1 diabetes is consistent with clinical evidence that there is a similar pathogenesis underlying the retinopathy in both type 1 and 2 diabetes [34]. As glycaemic control has little effect on the development and progression of neurodegenerative changes in these animals [35], the presence of both neuronal and vascular features may, by way of a targeted therapeutic manipulation, represent a robust platform allowing the interdependence or interaction of these distinct features to be understood. This has obvious implications for the evaluation of novel therapies.

We believe that BTBR *ob/ob* mice have an important role to play in future diabetic retinopathy research, including evaluation of the mechanisms that drive early neuronal and later retinal vascular changes. Our detailed characterisation of this mouse model should facilitate further examination of pathological mechanisms of type 2 diabetes diabetic retinopathy and the development of novel therapies for human diabetic retinopathy.

## Electronic supplementary material

ESM (PDF 54.3 mb)

## Data Availability

The data is available on request from the authors.

## Abbreviations

BRN3A: Brain-specific homeobox/POU domain protein 3A
GCL: Ganglion cell layer
GFAP: Glial fibrillary acidic protein
IB4: Isolectin B4
IBA-1: Ionised calcium-binding adapter molecule 1
IDA: Industrial denatured alcohol
INL: Inner nuclear layer
IPL: Inner plexiform layer
NFL: Nerve fibre layer
OCT: Optical coherence tomography
ONL: Outer nuclear layer
OPL: Outer plexiform layer
PECAM: Platelet endothelial cell adhesion molecule
RGC: Retinal ganglion cell
Rho-Con A: Rhodamine-conjugated concanavalin A
SD-OCT: Spectral domain optical coherence tomography
TEM: Transmission electron microscopy
T-PBS: Triton X-100 in PBS
