# Supplementary material for: BTBR ob/ob mouse model of type 2 diabetes exhibits early loss of retinal function and retinal inflammation followed by late vascular changes
Source: Diabetologia. 2018 Aug 9;61(11):2422–32. doi: 10.1007/s00125-018-4696-x (PMC6182653; doi:10.1007/s00125-018-4696-x)
Supplement: Supplementary file 1 — (PDF 54.3 mb) [file 125_2018_4696_MOESM1_ESM.pdf]

Electronic Supplementary figure 1

a

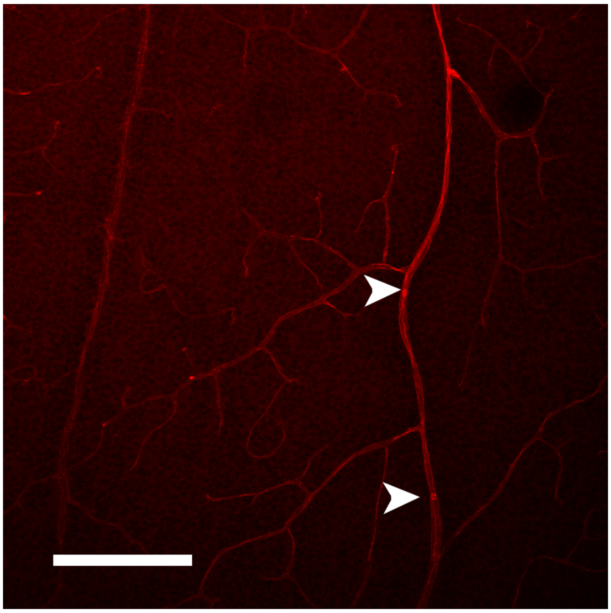


**ESM Figure 1**
(a) Representative immunofluorescence image of leukostasis where Rhodamine-Concanavalin A (Rho-Con A) labeled cells were found in retinal vessels (white arrowheads). Scale bar = 50μm.

# Electronic Supplementary figure 2

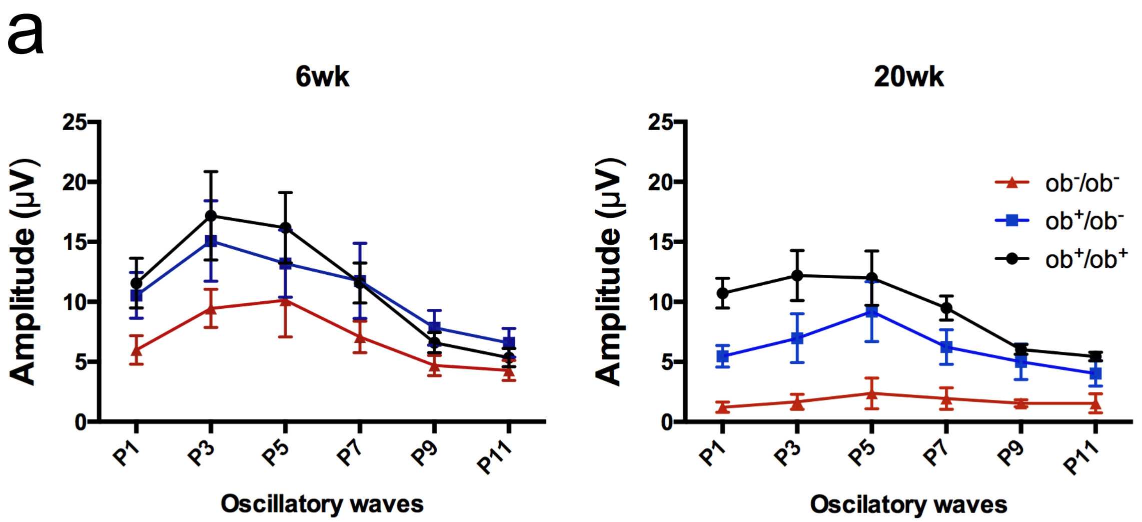


**ESM Figure 2**

Line graphs show oscillatory potential (OP) from scotopic electroretinograms recorded at maximum light level (2.87 log(cd x s/$m^2$)) of 6- and 20 weeks aged mice phenotypes $ob^+/ob^+$, $ob^+/ob^-$ and $ob^-/ob^-$. The first three oscillatory waves P1, P3 and P5 were found in the ascending b-wave. The OP wave P7 was in the descending b-wave, P9 and P11 were in the tail of the b-wave (which begins the c-wave). There were stronger responses in the recordings from 6-week-old retina. Also, the $ob^+/ob^+$ responses were the largest and the $ob^-/ob^-$ responses were weakest. (Two-way ANOVA, oscillatory wave $p<0.001$, phenotype $p<0.01$, n=4 eyes). This pattern was also seen in the 20-week-old recordings, however, they became weaker with aging. In addition, the differences between the phenotypes are wider. (Two-way ANOVA, oscillatory waves were not significant, phenotype $p<0.001$, n=4 eyes)

Electronic Supplementary figure 3

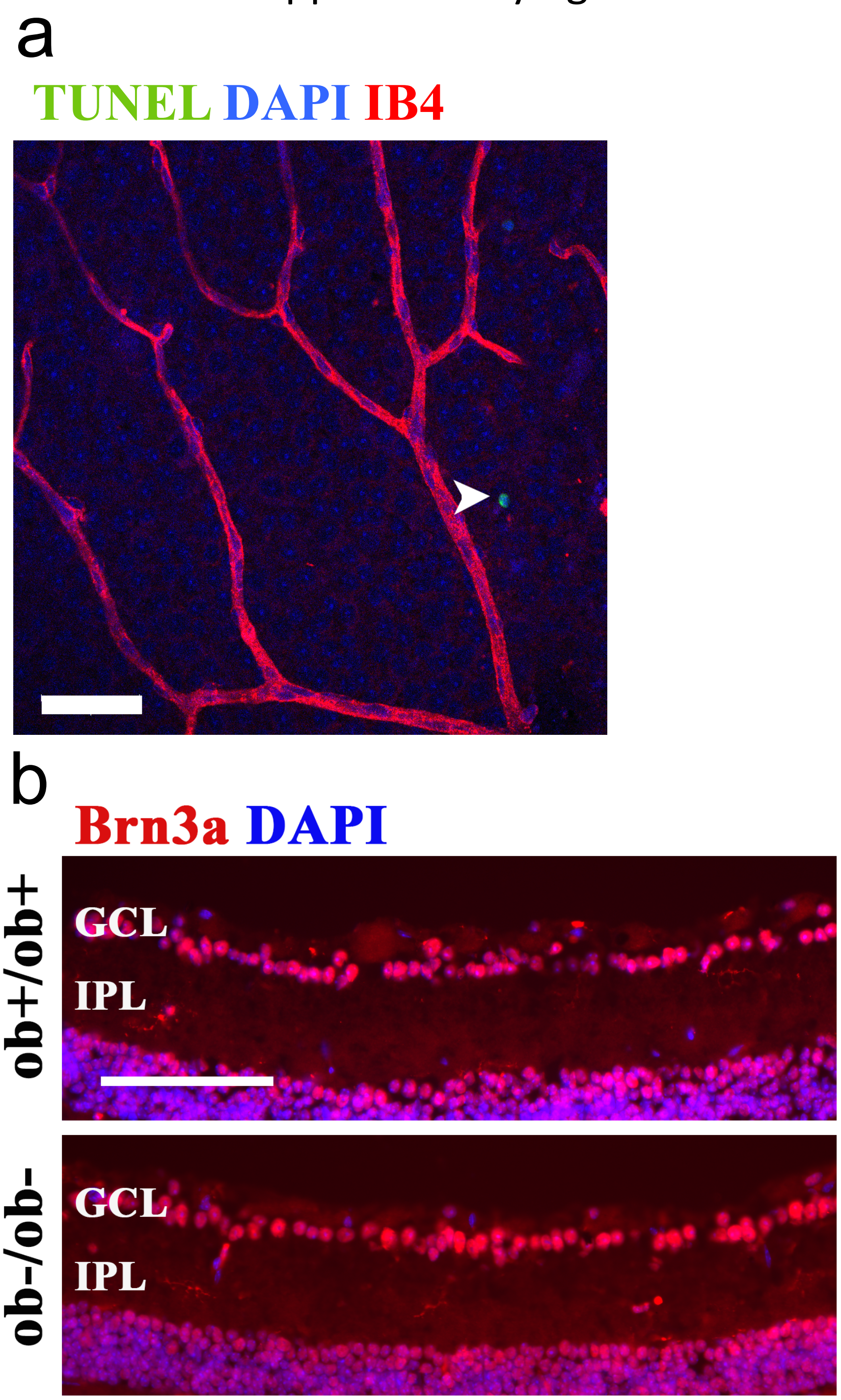


**ESM Figure 3**

(a) Representative immunofluorescence image of a TUNEL-positive ganglion cell (green). The nucleus (blue) was highlighted with the white arrowhead. The IB4 marked the blood vessels in the primary plexus (red). Scale bar = 50µm. (b) Representative immunofluorescence transverse section images of the inner retina in 6-week-old $ob^{+}/ob^{+}$ and $ob^{-}/ob^{-}$ mice. The nuclei (blue) marked with Brn3a (red) were ganglion cells. A smaller number of Brn3a expressing ganglion cells were found in the diabetic eye. Scale bar = 50µm. GCL Ganglion Cell layer; IPL Inner Plexiform layer.

Electronic Supplementary figure 4

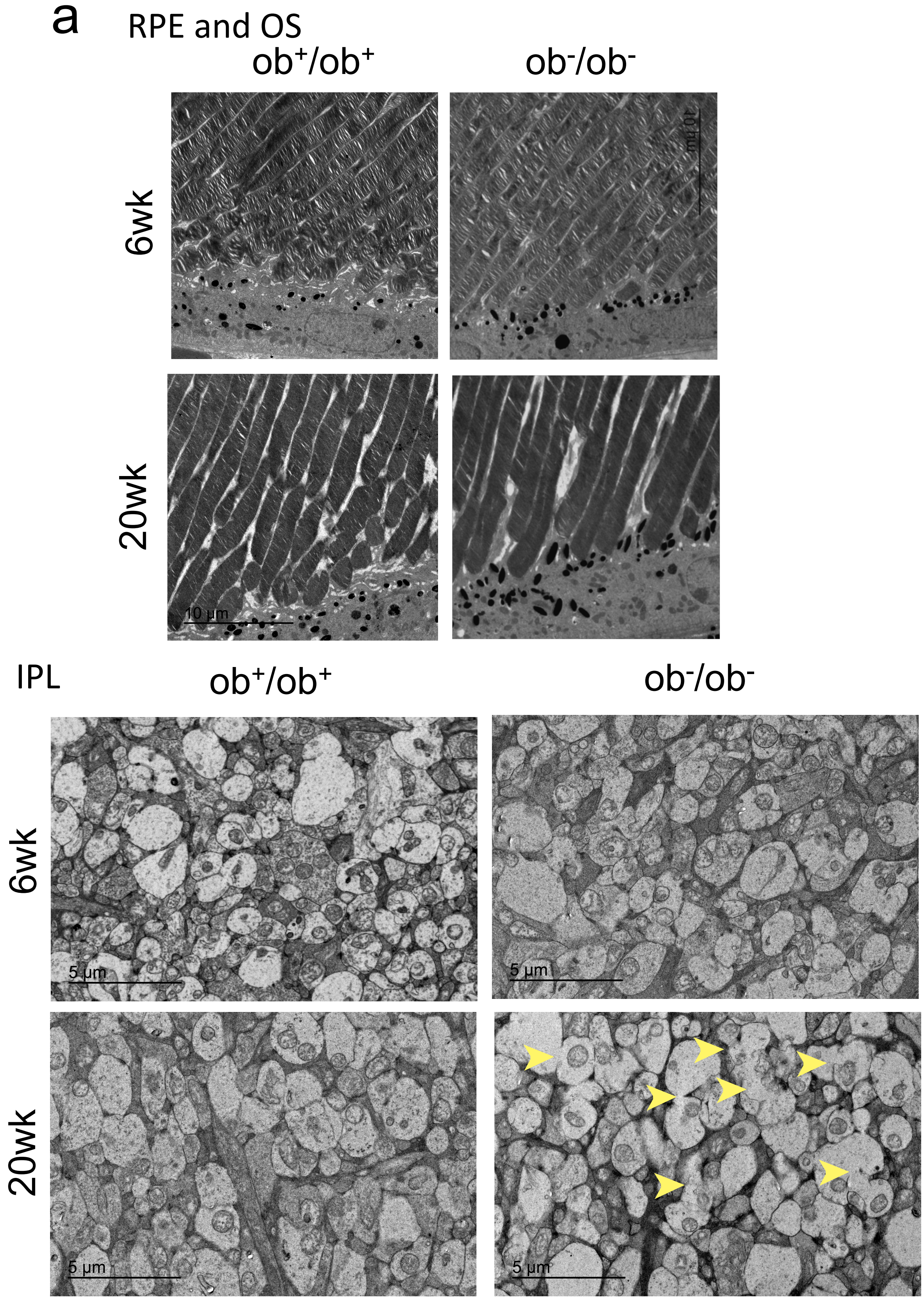

**ESM Figure 4**

(Top) Representative transmission electron micrographs (TEM) of the outer retina in 6- and 20-week-old $ob^+/ob^+$, $ob^-/ob^-$ mice, the retinal pigment epithelium (RPE) and outer segments (OS) appeared similar and intact. Scale bar = 10μm. (Bottom) Representative TEM of the inner plexiform layer (IPL) showed that in 20-week-old $ob^-/ob^-$, there were many axonal abnormalities where the axons merged together into larger processes. The yellow arrowheads points to absent axon cell membrane structures. Scale bar = 5μm.
